# Correction: Polydopamine-functionalized selenium nanoparticles as an efficient photoresponsive antibacterial platform

**DOI:** 10.1039/d3ra90039h

**Published:** 2023-04-26

**Authors:** Meng Sun, Ping Gao, Bao Wang, Xiangyang Li, Donghan Shao, Yan Xu, Leijiao Li, Yunhui Li, Jianwei Zhu, Wenliang Li, Yingxue Xue

**Affiliations:** a School of Chemistry and Environmental Engineering, Changchun University of Science and Technology Changchun 130022 China lileijiao@cust.edu.cn; b Zhongshan Institute of Changchun University of Science and Technology Zhongshan 528437 China; c Jilin Medical University Jilin 132013 China

## Abstract

Correction for ‘Polydopamine-functionalized selenium nanoparticles as an efficient photoresponsive antibacterial platform’ by Meng Sun *et al., RSC Adv.*, 2023, **13**, 9998–10004, https://doi.org/10.1039/D2RA07737J.

The authors regret the omission of a funding acknowledgement in the original article. This acknowledgement is given below.

We acknowledge the support from the Science and Technology Research Project of the Jilin Education Bureau (no. JJKH20220457KJ).

The Royal Society of Chemistry apologises for these errors and any consequent inconvenience to authors and readers.

## Supplementary Material

